# Lone-Pair-Like Interaction and Bonding Inhomogeneity Induce Ultralow Lattice Thermal Conductivity in Filled β-Manganese-Type Phases

**DOI:** 10.1021/acs.chemmater.2c00915

**Published:** 2022-05-27

**Authors:** Oleksandr Cherniushok, Raul Cardoso-Gil, Taras Parashchuk, Rafal Knura, Yuri Grin, Krzysztof T. Wojciechowski

**Affiliations:** † Thermoelectric Research Laboratory, Department of Inorganic Chemistry, Faculty of Materials Science and Ceramics, AGH University of Science and Technology, Mickiewicza Ave. 30, 30-059 Krakow, Poland; ‡ Max-Planck-Institut für Chemische Physik fester Stoffe, Nöthnitzer Str. 40, 01187 Dresden, Germany; § Department of Science, Graduate School of Science and Technology, Kumamoto University, 2 Chome-39-1 Kurokami, Chuo Ward, 860-8555 Kumamoto, Japan

## Abstract

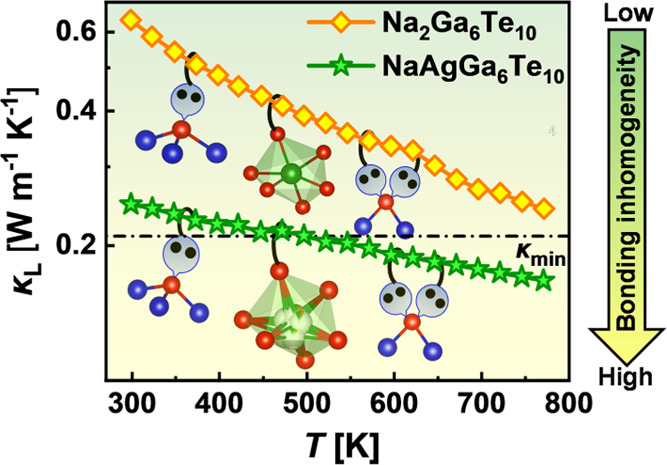

Finding a way to interlink heat transport with the crystal structure and order/disorder phenomena is crucial for designing materials with ultralow lattice thermal conductivity. Here, we revisit the crystal structure and explore the thermoelectric properties of several compounds from the family of the filled β-Mn-type phases *M*_2/*n*_^*n*+^Ga_6_Te_10_ (*M* = Pb, Sn, Ca, Na, Na + Ag). The strongly disturbed thermal transport observed in the investigated materials originates from a three-dimensional Te–Ga network with lone-pair-like interactions, which results in large variations of the Ga–Te and *M*–Te interatomic distances and substantial anharmonic effects. In the particular case of NaAgGa_6_Te_10_, the additional presence of different cations leads to bonding inhomogeneity and strong structural disorder, resulting in a dramatically low lattice thermal conductivity (∼0.25 Wm^–1^ K^–1^ at 298 K), being the lowest among the reported β-Mn-type phases. This study offers a way to develop materials with ultralow lattice thermal conductivity by considering bonding inhomogeneity and lone-pair-like interactions.

## 1 Introduction

The global increasing energy demand and depletion of natural resources are strong motivations for better exploitation of the available energy supplies and the development of new energy sources. Thermoelectric (TE) materials and devices are some of the research areas related to novel ecological energy sources due to the possibility of converting waste heat into electricity or vice versa.^1,2^ The main advantages of these devices relate to high reliability and a significantly long period of continuous operation.^3^ The energy conversion ability of the material can be determined by a dimensionless thermoelectric figure of merit, represented as *ZT* = *S*^*2*^*T*/ρ(κ_e_ + κ_L_), where *S* is the Seebeck coefficient, ρ is the electrical resistivity, *T* is the absolute temperature, and κ_e_ and κ_L_ are the electronic and lattice components of the thermal conductivity, respectively.^4^ A high power factor value (*S*^2^/ρ) and a thermal conductivity (κ) as low as possible over the operating temperature range are necessary for the high energy conversion efficiency.^5^ Because of the strong coupling among *S*, ρ, and κ_e_ via carrier concentration, band structure, and charge scattering, the individual optimization of these parameters is still a challenge.^6^ Consequently, the decisive requirement for a highly efficient thermoelectric material is a lattice thermal conductivity, κ_L_, that is as low as possible, which is the main focus of this work.

Many discussions related to the origins of low κ_L_ are available in the literature. The general statement of Goldsmid indicates that compounds with a high mean atomic weight tend to have a low lattice thermal conductivity because of the low frequency of lattice vibrations.^7^ Ioffe^8^ observed a decrease in thermal conductivity with an increase in polarity between the interacting atoms, and Spitzer^9^ showed a correlation between increasing coordination number in a crystal structure and decreasing lattice thermal conductivity. Recent publications show that the strength of chemical bonds is another crucial parameter that affects the value of lattice thermal conductivity.^3,10−12^ Spitzer also noticed that tetrahedrally coordinated binary zinc-blende-like compounds have strong covalent interactions associated with a very high lattice thermal conductivity. With the increasing polarity of their covalent bonds, the lattice thermal conductivity decreases; thus, A^II^B^VI^ compounds typically have a lower thermal conductivity (6–27 W m^–1^ K^–1^) than A^III^B^V^ semiconductors (35–90 W m^–1^ K^–1^).^9^ Moreover, A^IV^B^VI^ compounds with the NaCl structure and an octahedral coordination, due to the longer interatomic distances and weaker interaction between atoms, show a significantly lower lattice thermal conductivity (2–3.5 W m^–1^ K^–1^).^9,13,14^ New classes of extremely promising TE materials, i.e., superionic binary and ternary copper and silver chalcogenides, due to soft interactions of Cu^+^ (Ag^+^) ions with a liquid-like behavior, possess extremely low values of κ_L_ (0.5–0.9 W m^–1^ K^–1^ for binary Cu and Ag chalcogenides^15,16^ and 0.2–0.4 W m^–1^ K^–1^ for ternary argyrodites^17,18^), which makes these types of materials very interesting from the thermoelectric point of view. However, the main disadvantage of superionic thermoelectric materials is the low thermal stability due to cation migration, which causes structure degradation of the material.^19^

On the other hand, a large number of atoms in the unit cell *N* may also cause low lattice thermal conductivity,^20,21^ although this is not the rule.^22^ Increasing *N* in a material intuitively creates a more troublesome transport path for phonons, ultimately reducing κ_L_ by increasing phonon-scattering opportunities and slowing short-wavelength phonons.^10^ With the increasing number of atoms in the primitive unit cell, more optical branches appear in the phonon dispersion, and therefore, compounds with large unit cells should show low thermal conductivities.^11^ One of the newest concepts to explain the phonon transport in crystalline materials is based on the two-channel phonon transport approach, i.e., the phonon-gas channel (κ_pg_) and the diffuson channel (κ_diff_).^23^ While the phonon-gas channel is commonly believed to be the determinative factor of thermal conductivity, the diffuson channel becomes significant in very anharmonic and structurally complex materials that have many atoms per unit cell. It is expected, that with increasing crystal structure complexity, anharmonicity, defect concentration, and temperature, the phonon-gas channel will be suppressed and the diffuson channel will be promoted. Complex Zintl compounds have been explored as a new class of thermoelectrics,^24^ which form quite complex crystal structures with large unit cells. The complexity of the crystal structure and the huge unit cell of these compounds, despite the crystalline order, enable a very low lattice thermal conductivity (0.6 W m^–1^ K^–1^ at *T* = 300 K).^25^ Recently, Deng et al.^26^ showed that the increasing mismatch (δ) between the number of cations and anions in a structure is a simple indicator for lowering κ_L_ in ternary Cu- and Ag-based chalcogenides. Large δ indicates the presence of vacancies in certain crystallographic positions, which causes local bonding distortions including a change in bond lengths and angles. Later, systematic studies on single-crystalline materials revealed that the relation between the number of atoms in the primitive unit cell (which was considered a measure of structural complexity) and the thermal conductivity is not straightforward. Instead, the chemical bonding complexity was suggested to influence the lattice thermal conductivity.^22^ Therefore, to accommodate the diverse bonding conditions, a larger cell with more atoms and a lower symmetry tends to be adopted. According to the reasons for the low lattice thermal conductivity described above, we believe that a variety of new TE materials with ultralow κ_L_ could be developed further.

Aiming to explore new potential thermoelectric materials with ultralow lattice thermal conductivity, we found a group of ternary tellurides with a structural organization similar to the β-Mn structure.^27−29^ These filled β-manganese-type phases have the chemical composition *M*_2/*n*_^*n*+^*Tr*_6_^3+^*Q*_10_^2–^ (*M*^*n+*^: Li^+^, Na^+^, Ag^+^, Ca^2+^, Sn^2+^, Pb^2+^, Yb^2+^; *Tr*^3*+*^: Al^3+^, Ga^3+^, In^3+^; *Q*^2–^: Se^2–^, Te^2–^) and are characterized by close packings of *Q*_4_-tetrahedra, which is similar to the arrangement of manganese atoms in the cubic β-Mn. When *M*^*+*^ ions fill all available distorted octahedral voids, the *M*^*2+*^ ions occupy only half of them, and *Tr*^3+^ ions are distributed in an ordered wayover 15% of the tetrahedral voids. These filled β-manganese-type phases are characterized by large unit cells (*V* ≈ 3200–3600 Å^3^ ^27^) with heavy atoms, which predicts a low lattice thermal conductivity.

In this work, we performed a detailed study of the crystal structure and TE properties of several promising compounds for energy conversion: CaGa_6_Te_10_, SnGa_6_Te_10_, PbGa_6_Te_10_, Na_2_Ga_6_Te_10_, and NaAgGa_6_Te_10_. All investigated β-manganese-type phases possess very low lattice thermal conductivity, which mainly originates from the unique features of the crystal structure. Particularly, a three-dimensional Te–Ga network with stereochemically active lone-pair-like interactions on Te results in large variations in the Ga–Te and *M*–Te interatomic distances and large Grüneisen parameters reflecting lattice anharmonicity. The strongest disturbance of the thermal transport is found in the case of the mixed-cation compound NaAgGa_6_Te_10_, in which the bonding inhomogeneity is increased by different polarities of Ag–Te and Na–Te bonds. Considering filled β-manganese-type phases as examples, we show that bonding inhomogeneity and lone-pair-like interactions can be effectively used for the design of materials with ultralow lattice thermal conductivity.

## 2 Results and Discussion

### 2.1 Thermal and Microstructural Analyses

The polymorphic phase transition of PbGa_6_Te_10_ has been discovered and analyzed in our recent work.^30^ On the other hand, in the available literature^27,28^ on *M*Ga_6_Te_10_ compounds, no information is given about the polymorphic phase transition of other known compounds from this family.

The results of the DSC analysis of filled β-Mn-type phases are presented in the Supporting information, Figure S1. All studied ternary compounds show endothermic peaks in the range of ∼650–760 K. To verify the possible polymorphic phase transition, we additionally annealed samples of the ternary compounds at 823 K followed by quenching in an ice-water bath and performed PXRD analysis. All of the reflections were successfully indexed in the *R*32 space group. This means that in the earlier work of Deiseroth and Müller,^27^ the crystal structure of the high-temperature modifications of SnGa_6_Te_10_ and PbGa_6_Te_10_ with a rhombohedral *R*32 symmetry was proposed. In a further work, Deiseroth and Kienle^28^ described low-temperature modifications of SnGa_6_Te_10_ and PbGa_6_Te_10_ in the trigonal *P*3_1_21 or *P*3_2_21 space groups. However, the high-temperature modifications with disordered Pb^2+^ or Sn^2+^ cation sites crystallize in the rhombohedral *R*32 space group. Consequently, here, based on the crystallographic and thermal data, we report for the first time the discovered phase transitions for SnGa_6_Te_10_.

Figure S2 shows backscattered electron images of filled β-Mn-type phase samples after the SPS procedure. The microstructure images of pellets indicate good densification after the SPS procedure. The density of all samples after sintering was ≥95% of the crystallographic density. EDXS mapping of the mixed-cation NaAgGa_6_Te_10_ (Figure S2f) confirmed the homogeneous distribution of elements in the sample. The fractured surface of the NaAgGa_6_Te_10_ SPS-compacted pellet is shown in Figure S2g, and the higher-magnification image in Figure S2h evidences the polycrystalline nature of the sample.

### 2.2 Crystal Structure Determination

**Figure 1:**
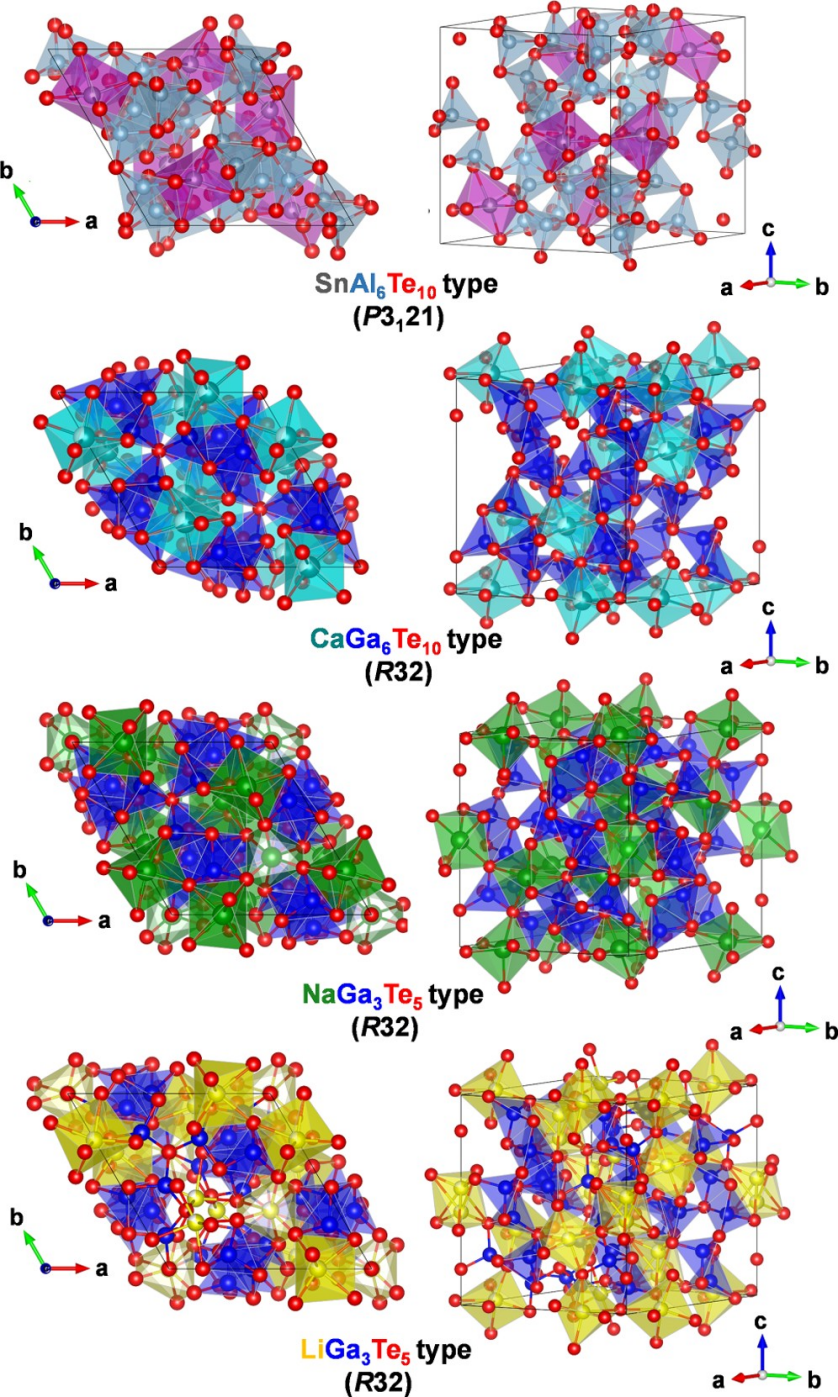
Crystal structure of the ordered and disordered prototypes of *M*_2/*n*_^*n*+^*Tr*_6_Te_10_ filled β-Mn-type phases.

Figure 1 shows the crystal structure of the reported ordered and disordered prototypes of *M*_2/*n*_^*n*+^*Tr*_6_Te_10_ filled β-Mn-type phases. These structural prototypes are characterized by a three-dimensional network formed by Ga and Te atoms, where different *M* cations can accommodate octahedral voids in an ordered or disordered way. For the crystal structure of CaGa_6_Te_10_, Cenzual et al.^31^ proposed a rhombohedral model, where Ca atoms occupy the octahedral 9*d* site in a disordered way with a site occupancy factor equal to 2/3 (Figure 1). The 3*b* octahedral site is unoccupied. Investigating the crystal structures of *M*Ga_6_Te_10_ (*M*: Pb^2+^, Sn^2+^), Deiseroth and Müller^27^ proposed a rhombohedral model (*R*32) with randomly disordered Pb^2+^ and Sn^2+^ ions (structure type CaGa_6_Te_10_). In a later work, based on single-crystal data, Deiseroth and Kienle^28^ presented derived models for the ordering of *M*^2+^ cations in the SnAl_6_Te_10_, SnGa_6_Te_10_, and PbGa_6_Te_10_ phases with the assumption of a trigonal symmetry (*P*3_1_21 or *P*3_2_21). These showed weak superstructure reflections, observed after annealing for several weeks. In the SnAl_6_Te_10_ structure, Sn^2+^ ions are distributed in an ordered way over the octahedral 6*c* position (Figure 1); however, the 3*a* and 3*b* octahedral sites remain empty.

In the case of *M*_2_Ga_6_Te_10_ compounds, the octahedral sites are occupied by *M*^+^ ions. According to the single-crystal data of Deiseroth and Kienle,^32^ Na_2_Ga_6_Te_10_ (or NaGa_3_Te_5_) crystallizes in an ordered rhombohedral structure (*R*32) with fully occupied 9*d* and 3*b* octahedral sites by Na^+^ ions (Figure 1). For Li_2_Ga_6_Te_10_ (or LiGa_3_Te_5_), the same authors proposed a disordered structural model, where Li^+^ ions occupy off-center positions in the 9*d* and 6*c* octahedral sites with site occupancy factor 4/5 for both sites (Figure 1).^33^ In the mixed-cation compound NaAgGa_6_Te_10_^34^, similar to Li^+^ in Li_2_Ga_6_Te_10_, Ag^+^ ions show positional disorder, however, in a 9*d* octahedral site. NaAgGa_6_Te_10_ can be treated as an intermediate phase between Na_2_Ga_6_Te_10_ and the high-pressure ionic conductor Ag_2_Ga_6_Te_10._^35^

**Figure 2:**
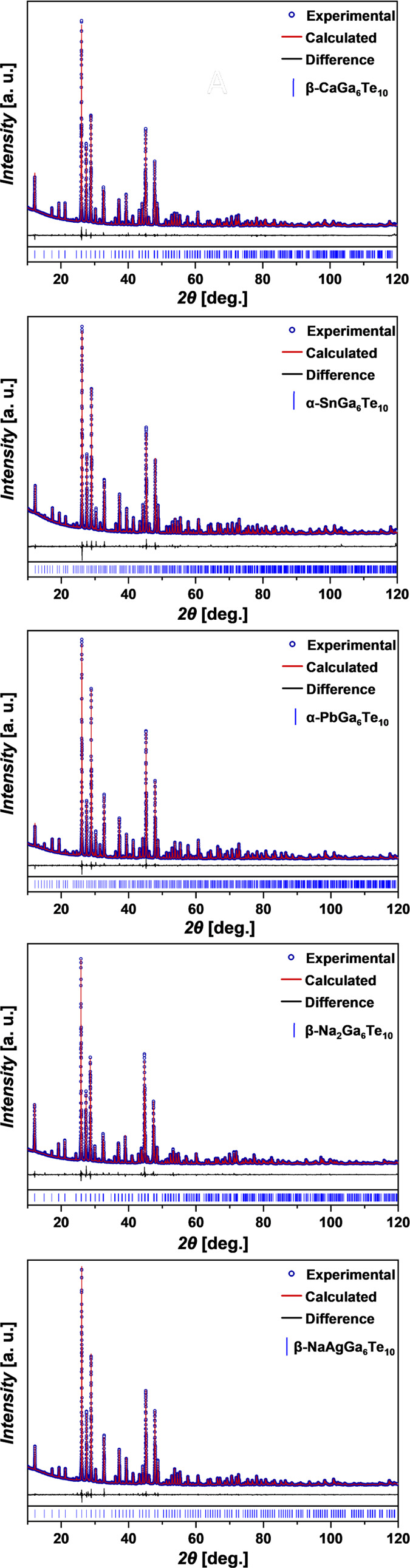
Powder XRD patterns of filled β-Mn-type phases.

Structural analysis of synthesized representatives of filled β-Mn-type phases CaGa_6_Te_10_, SnGa_6_Te_10_, PbGa_6_Te_10_, Na_2_Ga_6_Te_10_, and NaAgGa_6_Te_10_ was performed using powder X-ray diffraction (Figure 2). Crystallographic information and final refinement parameters are represented in Table 1, and atomic coordinates and isothermal displacement parameters are presented in Tables S1–S7.

**Table 1:** Crystallographic Information of Filled β-Mn-Type Phases

nominal composition	CaGa_6_Te_10_	SnGa_6_Te_10_	PbGa_6_Te_10_	Na_2_Ga_6_Te_10_	NaAgGa_6_Te_10_
structure type	CaGa_6_Te_10_	SnAl_6_Te_10_	SnAl_6_Te_10_	Na_2_Ga_6_Te_10_	NaAgGa_6_Te_10_
formula weight	1734.42	1813.05	1901.54	1740.32	1825.20
space group	*R*32 (No. 155)	*P*3_2_21 (No. 154)	*P*3_2_21 (No. 154)	*R*32 (No. 155)	*R*32 (No. 155)
*a* (Å)	14.4631(3)	14.439(1)	14.4784(2)	14.6184(7)	14.4997(6)
*c* (Å)	17.7950(4)	17.708(1)	17.7410(4)	17.7875(9)	17.7359(8)
unit cell volume (Å^3^)	3223.7(2)	3197.2(6)	3220.7(2)	3291.9(5)	3229.3(4)
*F* (000) (e)	4302.1	4509.2	4679.3	4394.1	4501.7
*Z*	6				
μ (cm^–1^)	1218.98	1297.49	1338.63	1181.69	1240.57
calculated density (g cm^–3^)	5.36	5.65	5.88	5.27	5.63
experimental density (g cm^–3^)	5.15	5.48	5.76	4.96	5.35
radiation, wavelength (Å)	Cu Kα, 1.54185				
*T* (K)	295				
data range 2θ (°)	10–120				
no. of reflections	596	1782	1796	616	607
no. of refined structure parameters	8	8	8	8	8
profile function	Pseudo-Voigt				
refinement mode	full profile				
*R*_*i*_	0.041	0.044	0.040	0.051	0.039
*R*_*p*_	0.024	0.025	0.023	0.032	0.022
*R*_wp_	0.036	0.038	0.033	0.048	0.033
goodness of fit	1.43	1.48	1.45	1.77	1.19

**Figure 3:**
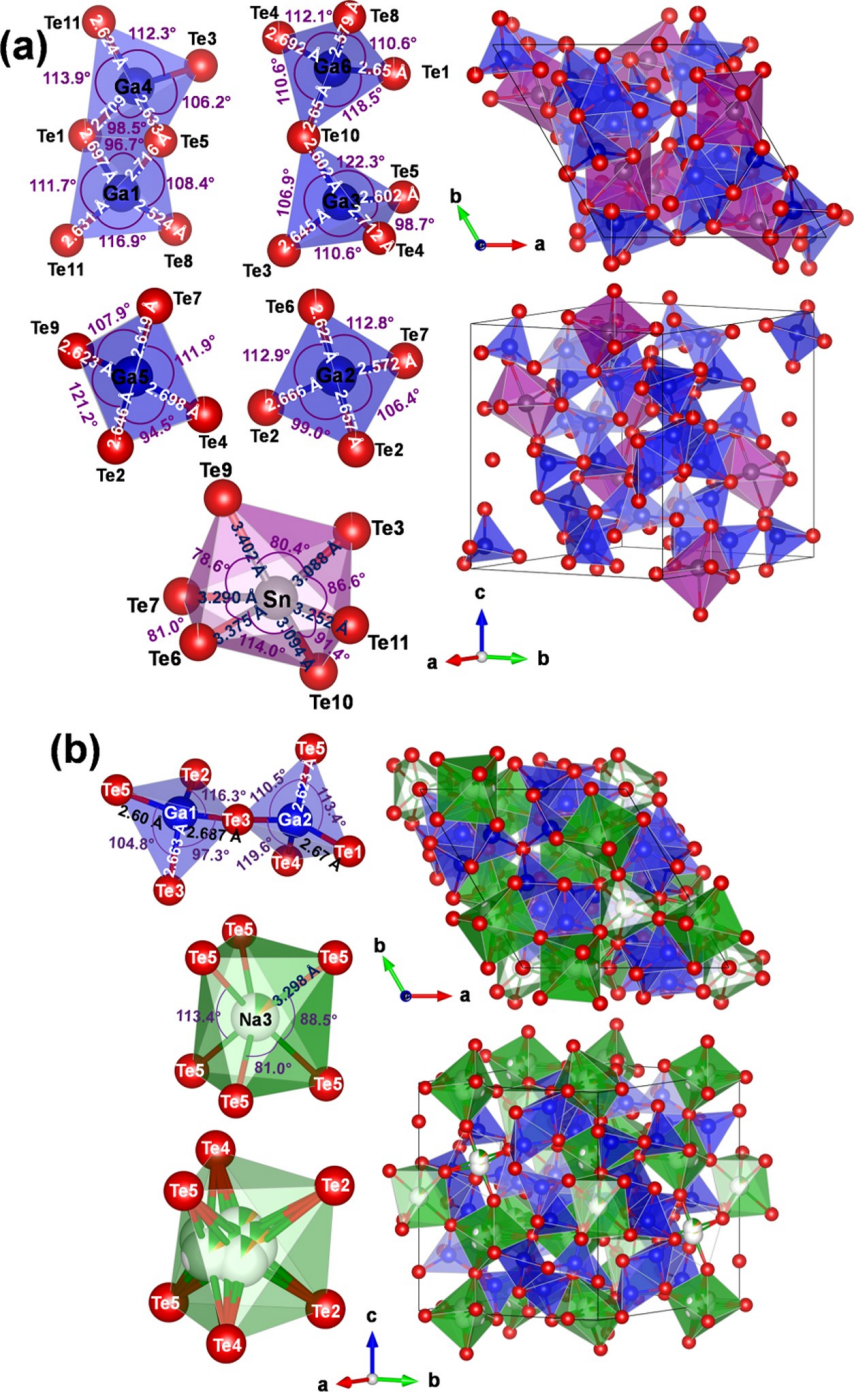
Crystal structures of ordered SnGa_6_Te_10_ (a) and disordered NaAgGa_6_Te_10_ (b) with corresponding coordination polyhedra. Represented structures are characterized by significant interatomic distance variations and bond angle distortions.

For the refinement of PbGa_6_Te_10_ and SnGa_6_Te_10_ crystal structures (Figure 3a), we used the models proposed by Deiseroth and Kienle^28^ with a trigonal symmetry (space group *P*3_1_21 or *P*3_2_21). All reflections were successfully indexed in the space group *P*3_2_21, and the crystal structure refinement yielded satisfactory residual values and atomic displacement parameters (ADPs). For CaGa_6_Te_10_, the acceptable residual values and atomic displacement parameters were obtained using the crystal structure model proposed by Cenzual et al.^31^ with the rhombohedral space group *R*32 as a starting model. The crystal structure refinement of Na_2_Ga_6_Te_10_ and NaAgGa_6_Te_10_ was performed using models in *R*32 as was first described by Deiseroth and Kienle.^32,34^ In the case of Na_2_Ga_6_Te_10_, satisfactory residual values and atomic displacement parameters were obtained for all atoms, except for Na in the 9*d* position, where the ADP was around two times higher than for Na in the 3*b* position (Table S4). This means that the proposed ordered model for the description of the crystal structure of Na_2_Ga_6_Te_10_ can be refined considering the possible disorder. Moreover, in NaAgGa_6_Te_10_, the resolution of the PXRD does not allow further study of the cation disorder. Therefore, in the case of the latter compounds, it was necessary to perform the structure refinement using single-crystal data.

As the microstructure analysis shows (Figure S2g,h), the prepared materials are polycrystalline. Nevertheless, it was possible to separate small μm-sized single-crystalline domains suitable for the single-crystal X-ray diffraction experiment. Obviously, because of the structural disorder, the final residual value of 0.063 for 1381 experimentally observed intensities was relatively high. Nevertheless, the obtained positions of Ga and Te are well in agreement with the previous crystal structure determinations for the compounds of the filled-β-Mn family.^27,32,34^ The calculation of the difference density (using only Ga and Te atoms) reveals the complex distribution of the latter and requires five positions for the appropriate description of its distribution (Figure 3b). Assuming the occupancy of all of these sites by sodium, it yields ca. 26 atoms per unit cell, which is much more than the 12 expected from the previous crystal structure determination^32^ and expected from the simple charge balance Na_2_^1+^Ga_6_^3+^Te_10_^2–^. Thus, the mixed occupation of all new positions by Na and Ag was derived by constraining the total number of atoms in them to 12 (Table S7, Supporting information). The so-obtained unit cell composition is Na_1.3_Ag_0.7_Ga_6_Te_10_, and the starting nominal composition of the sample is NaAgGa_6_Te_10_. While the cation position of the threefold axis (3*b* site 001/2) does not show disorder, the second cation position (9*d* site *x*00) seems to be strongly disordered and requires four partially occupied sites for its description. These findings in combination with the previous publications describing crystallographic disorder in the materials from the filled-β-Mn family show that the cation distribution in this atomic arrangement can vary, depending on the nature of the cation(s) *M* and the preparation conditions. The materials prepared in the present work reveal the strongest disorder among the known representatives of the *M*Ga_6_Te_10_ family.^32−35^

### 2.3 Chemical Bonding

**Figure 4:**
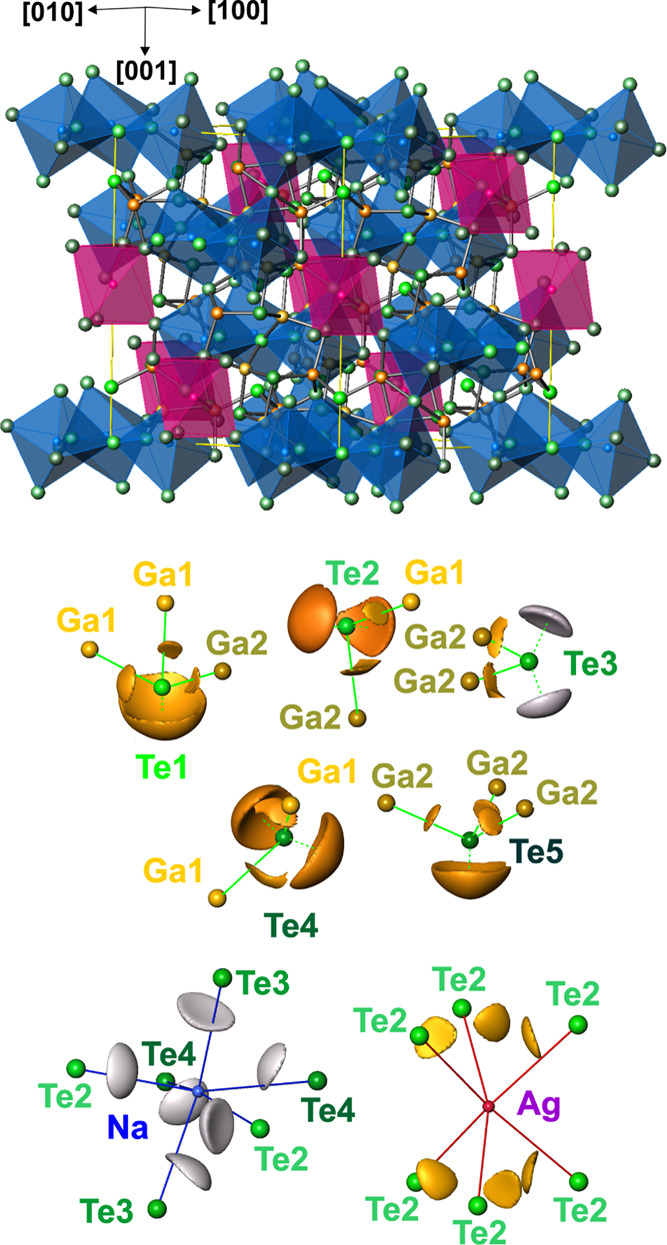
Crystal structure and bonding in Na_1.5_Ag_0.5_Ga_6_Te_10_: (top) Ga–Te network (gray lines) with the coordination polyhedrons of Ag (pink) and Na (blue); (middle) localization of ELI-D maxima around the Te atoms visualized by the isosurfaces of ELI-D for the 3-bonded Te1, 2-bonded Te2, Te3, Te4, and 3-bonded Te5, with the bold green lines showing bonding connections and the dashed green lines pointed out toward the lone-pair regions; (bottom) tellurium environment of the sodium (blue) and silver (pink) atoms with the isosurfaces of ELI-D visualizing the lone-pair-like (strongly polar) character of bonding in these regions.

From the analysis of interatomic distances, a three-dimensional network formed by Ga and Te atoms can be recognized in the crystal structure of Na_1.3_Ag_0.7_Ga_6_Te_10_ (gray in Figure 4, top). The distances between 2.58 and 2.67 Å are close to the sum of covalent radii (1.22 and 1.40 Å^36^). The atoms at the mixed (Na + Ag) occupied positions are separated from the Te ligands by much longer distances (mainly >2.98 Å). Thereby, the Te distances to the cationic site 3*b* (pink polyhedra in Figure 4, top) are markedly longer (3.30 Å) than the separations to the split positions around the 9*d* site (blue polyhedra in Figure 4, top). To find the bonding background for the distance distribution above, quantum mechanical calculations were performed on the ordered models with the compositions Na_1.5_Ag_0.5_Ga_6_Te_10_ and Na_0.5_Ag_1.5_Ga_6_Te_10_ (cf. experimental procedures).

The Te atoms turn out to play a decisive role in the chemical bonding and structural organization of the investigated material. There are two kinds of ELI-D maxima around the tellurium atoms (Figure 4, middle panel). The maxima of the first kind are located close to the Te–Ga contacts and represent the covalent bonds. The maxima of the second kind are located on that side of Te atoms where no Ga neighbors are in the vicinity. They visualize the lone-pair-like interactions in this region. Te1 and Te5 atoms have one such region and three bonding ones, i.e., (3b)Te, while Te2–Te4 atoms show two lone-pair-like regions and two bonding ones each, i.e., (2b)Te. According to the valence shell electron pair repulsion model,^37^ the volume demand of a valence shell electron pair (lone-pair) is higher in comparison with the bonding electron pair (stereochemical activity of lone pairs). Two lone-pair-like regions around the two-bonded Te2–Te4 require more volume in comparison with the ones at Te1 and Te5. This influences the coordination of the Ga1 and Ga2 atoms, in particular, the interatomic distances (on average, the distances between Ga and (3b)Te are longer than Ga–(2b)Te) and the Te–Ga–Te bond angles, which consequently deviate from the ideal tetrahedral values.

The use of the term “lone-pair-like” instead of “lone-pair” originates from the analysis of the interaction between Na and Ag on the one hand and their Te environments on the other. From the electron density–electron localizability indicator intersection procedure, the quantization of polar bonding can be obtained.^38,39^ Application of this technique to Na_1.5_Ag_0.5_Ga_6_Te_10_ reveals that—by the generally strong polarity of Na–Te and Ag–Te bonds—the contribution of the silver atoms to the lone-pair-like bonding is 2–3 times larger than that of the sodium atoms, and this is independent of which crystallographic sites (3*b* or 9*d*) silver is located. That means that the Ag–Te bonding is less polar than the Na–Te one. It was shown that the lattice thermal conductivity may be reduced by the appearance of different kinds of bonding in the material (bonding inhomogeneity).^22^ Thus, the different characters of the Na–Te and Ag–Te bonds may yield the difference in the thermal conductivity between the pristine Na_2_Ga_6_Te_10_ and its silver substitutional variety (cf. section 3.4).

### 2.4 Electronic Transport

**Figure 5:**
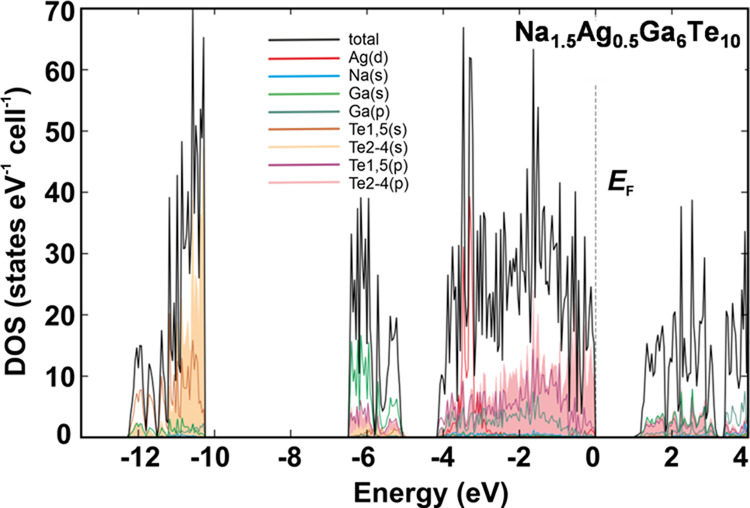
Calculated total electronic density of states for Na_1.5_Ag_0.5_Ga_6_Te_10_ with the contribution of relevant atomic states.

While the variation of chemical bonding may influence the lattice thermal conductivity of the *M*Ga_6_Te_10_ phases, it does not, in general, affect the electronic transport. Independent of the silver substitution, the calculated electronic density of state for Na_2–*x*_Ag_*x*_Ga_6_Te_10_ shows a clear gap (see Figure 5 for *x* = 0.5).

**Figure 6:**
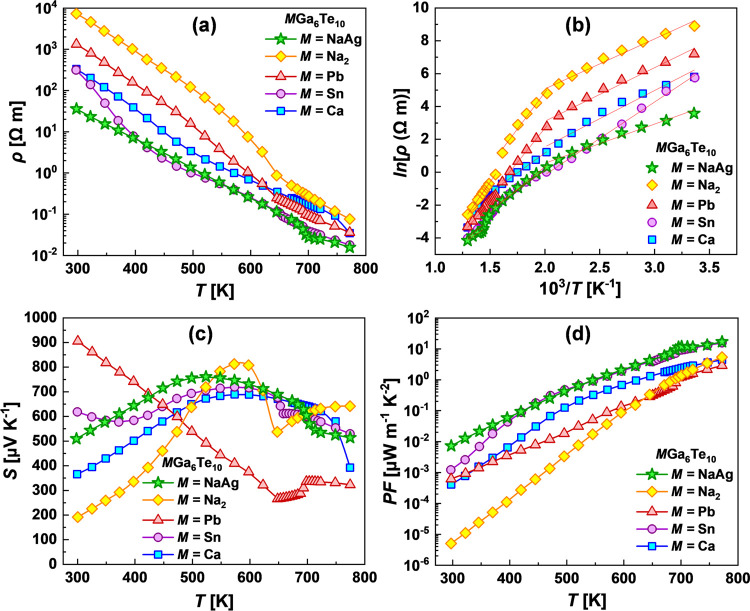
(a) Electrical resistivity, (b) Arrhenius plot of electrical resistivity, (c) Seebeck coefficient, and (d) thermoelectric power factor *PF* for filled β-manganese-type phases.

Figure 6 shows the electronic transport properties of *M*Ga_6_Te_10_ phases in the range of 298–773 K. The transport property measurements were performed with the step Δ*T* = 25 K; however, in the temperature region of endothermal effects registered by DSC, the measurements were carried out with the smaller temperature step Δ*T* ≤ 10 K to register possible unusual behavior of the Seebeck coefficient and electrical resistivity.

Figure 6a shows the electrical resistivity for filled β-manganese-type phases over the entire temperature range. The value of ρ is relatively high for the investigated compounds and decreases with increasing temperature, showing an intrinsic semiconducting transport behavior, agreeing with the calculated electronic density of states. Over the investigated temperature range, the pristine NaAgGa_6_Te_10_ and Na_2_Ga_6_Te_10_ samples show the lowest and the highest electrical resistivity curves, respectively. Inflections on the ρ(*Τ*) curves are observed in the phase transition region, as also noticed for PbGa_6_Te_10_ and SnGa_6_Te_10_. The values of ρ, being in the range of 3.6 × 10^1^ Ω·m (NaAgGa_6_Te_10_)—7.3 × 10^3^ Ω·m (Na_2_Ga_6_Te_10_) at 298 K, decrease to 1.6 × 10^–2^ Ω·m (NaAgGa_6_Te_10_)—7.6 × 10^–2^ Ω·m (Na_2_Ga_6_Te_10_) at 773 K. We also want to highlight the evident correlation between the endothermal effects of structural transitions registered on DSC and steplike changes of electrical resistivity in this range. The high resistivity of the studied materials is in agreement with the very low values of carrier concentration *n*_H_ in the range of 10^12^–10^14^ cm^–3^; however, the Hall measurements were too noisy to determine more precisely the values of *n*_H_.

The activation energies, estimated from the Arrhenius plot of the electrical resistivity (Figure 6b) for filled β-manganese-type phases, are given in Table S8. The values of *E*_a_ are varied from 0.4–0.8 eV in the low-temperature region to 0.8–1.65 eV in the high-temperature region. Among the measured samples, the lowest activation energies were estimated for NaAgGa_6_Te_10_ and SnGa_6_Te_10_, with the highest belonging to Na_2_Ga_6_Te_10_.

In the high-temperature region, the values of *E*_a_ are comparable to the reported band gaps for PbGa_6_Te_10_ and SnGa_6_Te_10_^40^ as well as in good agreement with the DFT-calculated band gap for NaAgGa_6_Te_10_. On the other hand, the low values of *E*_a_ at low temperatures might suggest the appearance of in-gap states or the presence of the hopping mechanism of electrical conductivity for the studied samples.

Figure 6c shows the temperature dependence of the Seebeck coefficient for filled β-manganese-type phases. All samples possess a positive Seebeck coefficient throughout the entire temperature range, indicating that holes are the dominant carriers. The Seebeck coefficient for all stoichiometric compounds, except for PbGa_6_Te_10_, shows a bell-shaped tendency with temperature, reaching a maximum of 700–800 μV K^–1^ at 500–600 K and also showing steplike changes on the *S*(*T*) during phase transitions in the high-temperature region.

Based on the measured *S* and ρ, the power factors (*PF* = *S*^2^/ρ) of the investigated filled β-manganese-type phases are calculated and presented in Figure 6d. Even if the Seebeck coefficients are very high for the studied compounds, due to high resistivities, the thermoelectric power factors are relatively low. Among all samples, the SnGa_6_Te_10_ and NaAgGa_6_Te_10_ have the highest *PF* in the range of ∼16–17 μW m^–1^ K^–2^ at 773 K.

**Figure 7:**
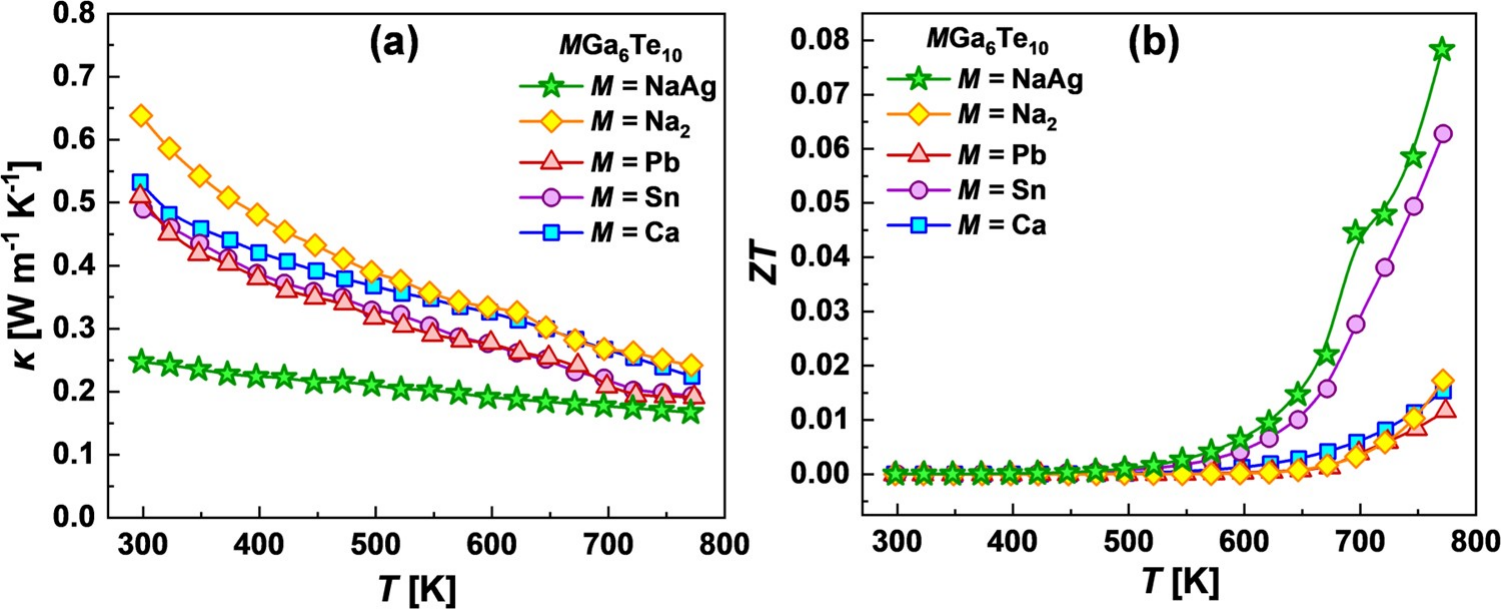
(a) Thermal conductivity and (b) dimensionless thermoelectric figure of merit *ZT* for filled β-manganese-type phases.

The *M*Ga_6_Te_10_ (*M* = Pb, Sn, Ca, Na_2_) compounds show very low decreasing dependences of κ(*Τ*) (Figure 7a) in the range of 0.49–0.64 W m^–1^ K^–1^ at 298 K to 0.19-0.24 W m^–1^ K^–1^ at 773 K. The decreasing tendency of the thermal conductivity can also be observed in series Na_2_Ga_6_Te_10_–CaGa_6_Te_10_–SnGa_6_Te_10_–PbGa_6_Te_10_, which correlates well with the increasing atomic weight of *M* cations (i.e., heavier atoms cause a lower thermal conductivity). It should be pointed out that a drop in thermal conductivity in the region of endothermal effects registered on DSC curves above 600 K appears, most probably, due to the latent heat of phase transitions, which is not taken into account in the Dulong–Petit heat capacity.^41^ Therefore, we should keep in mind that the thermal conductivity in the region of the phase transition can be somewhat underestimated. The most intriguing finding of this work is that the mixed-cation compound NaAgGa_6_Te_10_ shows dramatically lower thermal conductivities of about 0.25 W m^–1^ K^–1^ at 298 K and 0.17 W m^–1^ K^–1^ at 773 K, which are among the lowest values observed in crystalline materials reported to date. The almost temperature-independent thermal conductivity for the mixed-cation sample can be related to the heat transport preferably through the diffuson channel.^23^ The diffuson-provoked phonon transport, which is highly expected for the disordered systems with high anharmonicity, approaches the thermal conductivity to a constant value at high temperatures.^42^ On the other hand, the most realistic reason for such a strong reduction of κ compared to Na_2_Ga_6_Te_10_ can be explained in terms of the crystal chemistry uniqueness of NaAgGa_6_Te_10_ and will be discussed in more detail in the next section.

The dimensionless TE figure of merit (*ZT*) of the filled β-manganese-type samples is calculated based on the measured *S*, ρ, and κ and shown in Figure 7b. Due to the improved power factor up to 17 μW m^–1^ K^–2^ (Figure 6d), as well as the ultralow κ, the *ZT* value was improved by about 4.5 times (Figure 7b) for NaAgGa_6_Te_10_ in comparison with the Na_2_Ga_6_Te_10_ compound. Moreover, the lowest resistivity, as well as the ultralow lattice thermal conductivity of NaAgGa_6_Te_10_, makes this compound the most interesting for further investigation concerning tuning of the carrier concentration, which can significantly improve the TE performance.

### 2.5 Origins of Low Thermal Conductivity

Thermal transport in crystalline materials mainly originates from phonon–phonon scattering, point defects, micro- and nanoinclusions, grain boundaries, and crystal structure complexities.^43^ Finding the interlink between crystal structure and thermal conductivity has become one of the main topics of interest in modern solid-state physics.^10,44^ One of the parameters that connect the particularities of crystal structure and thermal conductivity is the Grüneisen parameter γ. The local estimation of the Grüneisen parameter is possible using the X-ray absorption fine structure (XAFS) method,^45^ and the average value of γ can be easily determined from ultrasonic measurements.^10,46^ Although such an approach does not provide full insight into the origin of the lattice thermal conductivity, it gives essential information about the dominant mechanism of thermal transport.

In our recent work,^30^ we showed that the point defects in Pb positions disturb the phonon transport in the PbGa_6_Te_10_ structure. Particularly, the thermal conductivity was lowered from 0.52 W m^–1^ K^–1^ for the stoichiometric composition PbGa_6_Te_10_ to 0.38 W m^–1^ K^–1^ for the cation-deficient Pb_0.9_Ga_6_Te_10_ sample at 298 K. However, the relatively small modification of the nominal composition results in the remarkable lowering of the thermal conductivity. Therefore, this work aims to understand the influence of different *M* atoms as well as partial occupancy of the atom positions on the thermal conductivity of *M*Ga_6_Te_10_. Keeping this in mind, we combined the ultrasonic measurements of longitudinal and transverse sound velocities at room temperature with the theoretical calculations based on the Callaway approach and with the analysis of the crystal structure features for the investigated *M*Ga_6_Te_10_ compounds.

The measured values of the longitudinal ν_*l*_ and transverse ν_t_ velocities and the results of the calculations of Debye temperatures Θ_D_, the Poisson ratio ν, the Grüneisen parameter γ, the bulk modulus *B*, Young’s modulus *E*, phonon mean free path *l*_ph_, and the minimum thermal conductivity κ_glass_ and κ_diff_ for filled β-manganese-type phases are shown in Table 2.

**Table 2:** Elastic and Thermal Transport Properties of Filled β-Manganese-Type Phases

compound	*v*_*l*_, m s^–1^	v_t_, m s^–1^	*v*_m_, m s^–1^	Θ_D_, K	ν	γ	*B*, GPa	*E*, GPa	*l*_ph_, Å	κ_glass_, W m^–1^ K^–1^	κ_diff_, W m^–1^ K^–1^
CaGa_6_Te_10_	2749	1668	1843	171.3	0.21	1.32	19.8	34.6	6.18	0.34	0.21
SnGa_6_Te_10_	2730	1640	1814	170.7	0.22	1.37	21.6	36.7	6.19	0.34	0.21
PbGa_6_Te_10_	2633	1585	1753	164.0	0.22	1.35	20.6	35.2	6.82	0.32	0.20
Na_2_Ga_6_Te_10_	2799	1669	1847	169.3	0.22	1.38	20.4	33.8	7.00	0.35	0.22
NaAgGa_6_Te_10_	2720	1577	1750	161.9	0.25	1.48	21.8	33.2	3.01	0.34	0.21

The calculation procedure for the elastic and thermal transport parameters can be found in the Supporting Information (eqs S1–S11). The obtained low values of the Debye temperature are typical for materials with weak interatomic interactions.^47^ On the other hand, the estimated Grüneisen parameters γ ∼ 1.32–1.48 are much higher compared to the diamond-like compounds with a tetrahedral coordination, which usually show γ ∼ 0.5–0.7.^10^ As the Grüneisen parameter is the direct measure of lattice anharmonicity^48^ (which is defined as a property of lattice vibrations governing how they interact and how well they conduct heat^49^), a strong lattice anharmonicity for *M*Ga_6_Te_10_, especially in the case of the mixed-cation compound NaAgGa_6_Te_10_ (where γ shows the highest value (Table 2)), is expected. It is worth mentioning that the estimation of the Gruneisen parameter from elastic properties may bring some errors depending on the model used for calculations.^50^ Considering the ratio of the longitudinal and transverse speeds of sound for the investigated compounds , in this work, we used the average speed of sound described by Anderson.^51^

The investigated materials are characterized by low values of Young’s modulus (*E* ≈ 33–37 GPa), also indicating a weak interaction between atoms in the materials. Such low values of Young’s modulus are comparable to Bi_2_Te_3_-based alloys,^10^ which contain weak van der Waals interactions in the structure. Low values of mean sound velocities result in a very short phonon mean free path of ∼3–7 Å, which is 2–5 times shorter than the lattice parameters. Such a low mean free path can also be connected with the lattice anharmonicity, as was recently shown by Lu et al.^52^ for PbTe. In the case of the investigated β-Mn-type compounds, the presence of lattice anharmonicity is connected with the lone-pair-like interactions, as was derived from our chemical bonding analysis. The lattice anharmonicity created by lone-pair interactions was also reported as the main cause of the low lattice thermal conductivity for pnicto-chalcogenide-based compounds.^44^

To understand the thermal transport in more detail, we also calculated the “minimum thermal conductivity” using two different approaches. All samples possess a lower κ than Cahill’s formulation of the glass limit for the thermal conductivity κ_glass_ based on the maximum phonon-scattering approach.^53^ Consequently, for estimation of a minimum of the thermal conductivity κ_diff_, the diffuson-based model adapted for disordered systems was employed.^42^ The majority of the investigated samples approach a minimum thermal conductivity κ_diff_ at high temperatures; however, the NaAgGa_6_Te_10_ samples even cross this minimal threshold, which makes confusing the understanding of the thermal transport in this material using conventional phonon transport theories.

**Figure 8:**
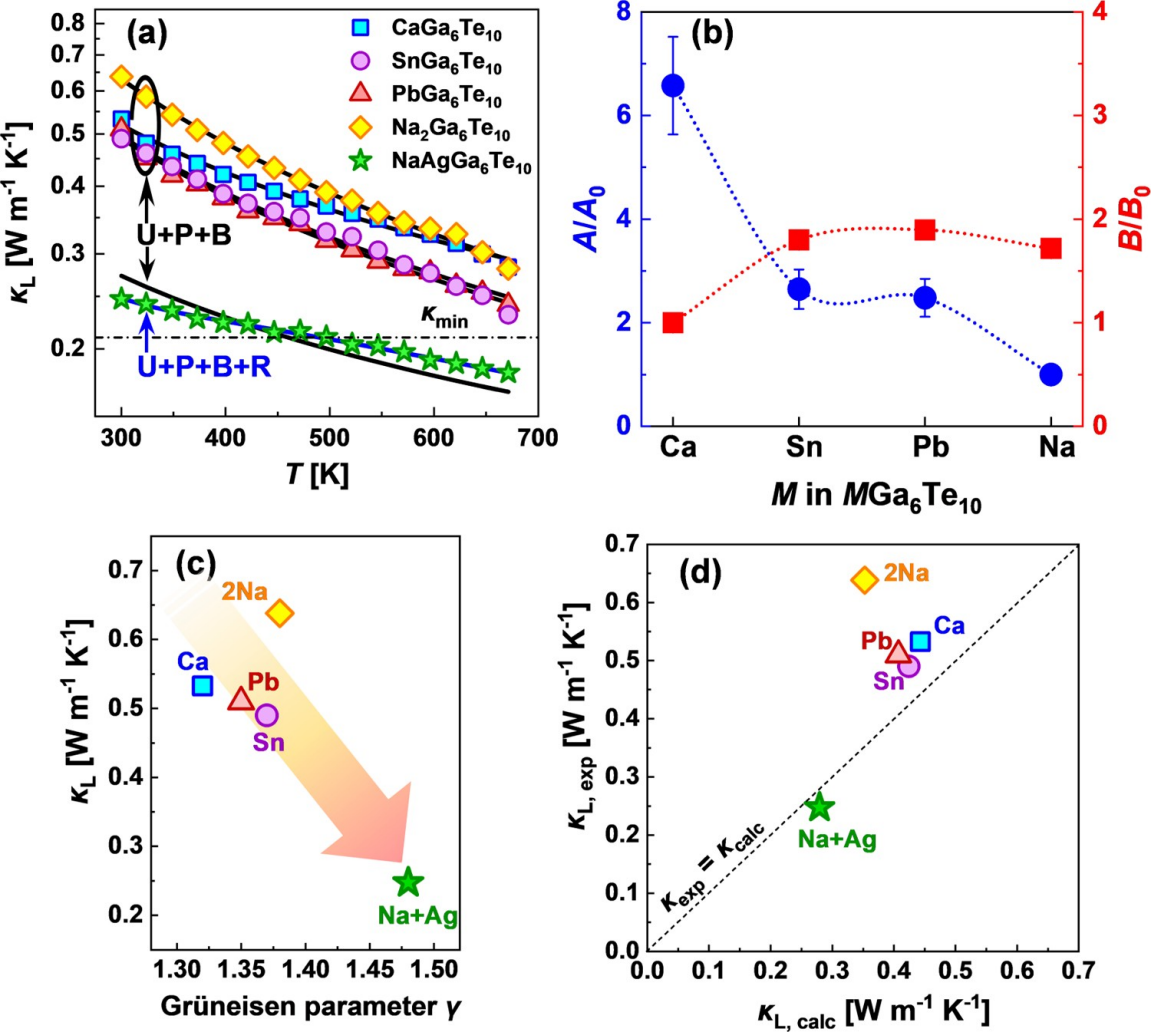
(a) Lattice thermal conductivity for the studied filled β-manganese-type phases; lines correspond to the calculations using the Callaway approach. Here, the U, P, B, and R represent Umklapp scattering, point-defect scattering, grain boundary scattering, and phonon resonance scattering, respectively. The κ_min_ indicates the theoretical minimum lattice thermal conductivity of NaAgGa_6_Te_10_. (b) Fitting parameters *A* and *B*, which were used for the calculation of lattice thermal conductivity by the Callaway approach; parameter *A* quantifies the strength of phonon scattering on point defects, and parameter *B* denotes the three phonon Umklapp scattering processes. (c) Experimental lattice thermal conductivity κ_L_ versus Grüneisen parameters γ determined from ultrasonic measurements. (d) Dependence between experimental and calculated (using eq S9) lattice thermal conductivities for the investigated samples.

Taking into account the high resistivity of the investigated samples, the electronic part of thermal conductivity is negligible, and we assumed that the total thermal conductivity contains only the lattice contribution κ_L_ (Figure 8). To further understand the low values of the lattice thermal conductivity for the investigated compounds, we used the Callaway approach^54^
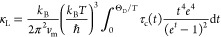
1

Within this approach, the phonon relaxation time (τ_c_) can be calculated using contributions related to scattering on phonon–phonon Umklapp processes, point defects, and grain boundaries (eqs S12–S14).

All investigated lattice thermal conductivity dependences were reasonably well-fitted by the Callaway model (Figure 8a). For single-cation β-manganese-type phases, it was enough to employ a model that accounts for the phonon Umklapp scattering + point defects + grain boundaries (black line). As the effect of the grain boundaries can be assumed to be the the same for all investigated specimens, we focused on the analysis of the fitting parameters *A* and *B* (Figure 8b), which quantify the strength of phonon scattering on point defects and phonon–phonon Umklapp scattering processes, respectively. The highest *A* parameter for CaGa_6_Te_10_ (Figure 8b) can be explained in terms of crystal chemistry because this compound crystallizes in a structure (*R*32 space group) with 2/3 occupancy of the 9*d* site by Ca^2+^. This feature may cause stronger point-defect scattering, in contrast to ordered SnGa_6_Te_10_ and PbGa_6_Te_10_, where this influence is weaker. In line with the mentioned reasons, Na_2_Ga_6_Te_10_ has even weaker point-defect scattering because all of its octahedral voids are filled by Na^+^ ions. The parameter *B* was found to be comparable for single-cation filled β-Mn-type phases, suggesting a similar effect of the phonon–phonon scattering for all four representatives. We would like to point out that the three phonon-scattering mechanisms, i.e., the point defects, Umklapp, and grain boundaries, were sufficient to reliably fit the experimental results of the lattice thermal conductivity for single-cation filled β-Mn-type phases. It is however possible that other phonon-scattering effects (e.g., phonon scattering by structural disorder^55^ or phonon resonance scattering^56^) are present, but their contribution is relatively small, compared to the three listed above.

In the case of the mixed-cation compound, taking into account only Umklapp, point defect, and grain boundary scattering does not give sufficient agreement with the experimental trend of the lattice thermal conductivity (black line in Figure 8a). To obtain a reasonably good fit for mixed-cation NaAgGa_6_Te_10_, the phonon resonance scattering (blue line in Figure 8a) was additionally employed for the calculations of the phonon relaxation times (eq S15).^56^ As was reported by Xie et al. for Cs-based halide perovskites,^57^ phonon resonance scattering has an essential effect on thermal transport due to the coupling between the low-frequency optical phonons and acoustic phonons, which originates from the weak chemical bonding between off-centered cations and halogen anions. Such a situation is highly probable in the case of the strongly disordered structures with bonding inhomogeneities, low Debye temperatures, and low phonon velocities. A similar set of properties was also discussed above for the filled β-Mn-type phases, especially in the case of mixed-cation NaAgGa_6_Te_10_, where disorder around the 9*d* cation site, a stronger bonding inhomogeneity, a lower Debye temperature, and a lower phonon velocity than in single-cation compounds were observed. Moreover, coupling between the low-frequency optical phonons with heat-carrying acoustical phonons was indicated by Cheng et al.^58^ for the PbGa_6_Te_10_ representative of filled β-Mn-type phases using DFT calculations.

The necessity to implement the additional terms to the Callaway model may be an indicator of the nonacceptance of the classical heat transport approach in this case. The two-channel (diffuson and phonon gas) thermal transport can be very helpful here.^23^ While the diffuson channel is suppressed in the single-cation compounds, the phonon-gas channel will be dominated. On the other hand, in the case of the cation-disorder compounds, the diffuson channel will be promoted. This can also lead to the almost temperature-independent thermal conductivity for NaAgGa_6_Te_10_.

According to the chemical bonding analysis, the presence of the stereochemically active lone-pair-like bonds on tellurium in the Ga–Te framework may induce lattice anharmonicity,^44^ which is reflected in the high Grüneisen parameters for our samples. Moreover, as calculated from ultrasonic measurements, the Grüneisen parameters correlate well with lattice thermal conductivities, as depicted in Figure 8c,d.

From the analysis of the chemical bonding above, the generally low lattice thermal conductivity of the filled β-manganese-type phases can be understood as a consequence of the coexistence of different types of bondings in these materials (bonding inhomogeneity^22,59,60^). The situation of the *M*Ga_6_Te_10_ phases is very similar to that of the intermetallic clathrates, where the covalent bonds in the three-dimensional framework formed by transition-metal and group 13 or group 14 elements are combined with complex interactions between the framework and filler atoms.^61^ The difference in the lattice thermal conductivity between Na_2_Ga_6_Te_10_ and NaAgGa_6_Te_10_ (and also in general between single-cation and two-cation compounds) can be understood by the stronger bonding inhomogeneity in NaAgGa_6_Te_10_: besides a variety of the Na–Te bonds due to the disorder around the 9*d* site, which is common for Ag-free and Ag-containing materials, less polar Ag–Te bonds appear in NaAgGa_6_Te_10_. This observation goes along with the case of the intermetallic clathrates, where the additional type of chemical bonding between the filler atoms and the more electronegative component in the framework position reduces the lattice thermal conductivity.^62−64^ The lone-pair-like interactions and bonding inhomogeneities are the main reasons for a dramatic reduction of the lattice thermal conductivity in the investigated mixed-cation-filled β-manganese-type phases.

## 3 Conclusions

The structural and thermoelectric properties of the filled β-manganese-type phases *M*_2/*n*_^*n*+^Ga_6_Te_10_ (*M* = Pb, Sn, Ca, Na, Na + Ag) were characterized aiming to understand and influence the heat transport. All investigated members of this family possess a very low thermal conductivity, mainly due to lone-pair-like interactions on Te. The most intriguing finding is that the mixed-cation compound NaAgGa_6_Te_10_ shows extremely low thermal conductivity, about 0.25 W m^–1^ K^–1^ at 298 K, decreasing to 0.17 W m^–1^ K^–1^ at 773 K, which is among the lowest values observed to date in crystalline materials. This feature can be understood due to the synergistic effect of the disorder around the 9*d* site and the stronger bonding inhomogeneity caused by the appearance of Na–Te bonds and Ag–Te bonds with different polarities in NaAgGa_6_Te_10_. Such a property results in troublesome thermal transport in this material and can be described by resonance phonon scattering as it was derived from Callaway’s analysis. Due to the improved power factor as well as the ultralow κ, the *ZT* value was enhanced by about 4 times for NaAgGa_6_Te_10_ in comparison with the Na_2_Ga_6_Te_10_ compound. Furthermore, the large thermoelectric potential of the investigated compounds is related to the high Seebeck coefficient in the range of 600–800 μV K^–1^.

This work shows that the analysis of chemical bonding offers an explanation of the low lattice thermal conductivity observed in complex chalcogenides and opens a new route for the design of novel materials with ultralow lattice thermal conductivity for various applications, including thermoelectrics and thermal barrier coatings.

## 4 Experimental Section

### 4.1 Preparation

The synthesis of all samples was carried out in graphite-coated (to avoid possible reaction with SiO_2_) quartz ampoules, evacuated to a residual pressure of 10^–5^ mbar, and sealed with an oxygen–hydrogen burner flame. Prior to synthesis, the ampoules were subjected to rigorous cleaning, which included washing in a 1HNO_3_:3HCl acid mixture and frequent flushing with distilled water and isopropanol for final drying. Polycrystalline samples were synthesized in a muffle furnace by reacting the elements Ca (Alfa Aesar, 99.99%), Sn (Alfa Aesar, 99.999%), Pb (Alfa Aesar, 99.999%), Na (Alfa Aesar, 99.99%), Ag (Alfa Aesar, 99.999%), Ga (Alfa Aesar, 99.9999%), and Te (Alfa Aesar, 99.999%) at 1223 K for 5 h. Then, the furnace in the upright position was cooled in the inertial mode to room temperature. The resultant dark-gray and brittle ingots were ground to powder, cold-pressed, and annealed for 200 h at 873 K in evacuated quartz ampoules. After the annealing process, the samples were cooled in the inertial mode to room temperature.

Synthesized materials were crushed into fine powders manually in an agate mortar and then densified using a spark plasma sintering (SPS) laboratory-made apparatus at 773 K for 20 min in a 12.8 mm-diameter graphite mold under an axial compressive stress of 45 MPa in an argon atmosphere. The heating and cooling rates were 50 and 20 K/min, respectively. The obtained dense pellets (*d* ≥ 95% of the crystallographic density) with a diameter of 12.8 mm and height ∼2 mm were then polished for transport property measurements.

### 4.2 Structural and Thermal Analyses

Phase identification was performed with a Bruker D8 Advance X-ray diffractometer using Cu *K*α radiation (λ = 1.5418 Å, Δ2θ = 0.005°, 2θ range 10–120°) with Bragg–Brentano geometry. Rietveld refinement of the crystal structure was performed using the WinCSD program package.^65^

Thermal analysis of the different samples was performed on a differential scanning calorimetry equipment (NETZSCH DSC 404 F3 *Pegasus*) using a sample mass of ∼10 mg in Al crucibles with a lid using a heating rate of 10 K/min under a helium flow.

For SEM and EDXS analyses, samples were embedded in conductive resin and subsequently polished finely using 0.1 μm diamond powder in a slurry. The analysis of the chemical composition was performed using scanning electron microscopy (JEOL JSM-6460LV scanning electron microscope) equipped with energy-dispersive X-ray spectroscopy.

### 4.3 Electrical and Thermal Transport Properties

The Seebeck coefficient α and electrical resistivity ρ were measured by commercial apparatus NETZSCH SBA 458 *Nemesis*. Measurements were spent in an argon flow in the temperature range of 298–773 K. Thermal diffusivity α_D_ was measured on a NETZSCH LFA 457 equipment, and the specific heat capacity *C*_p_ was estimated from the Dulong–Petit limit. The samples were first spray-coated with a thin layer of graphite to minimize errors from the emissivity of the material and laser beam reflection caused by a shiny pellet surface. Thermal conductivity was calculated using the equation κ *= dC*_*p*_α_D_, where *d* is the density obtained by the Archimedes principle at the pellets from SPS. The uncertainty values of the Seebeck coefficient and electrical conductivity measurements were 7 and 5%, respectively, whereas that of thermal diffusivity measurements was 3%. The combined uncertainty for the determination of the thermoelectric figure of merit *ZT* was ∼20%.^66^ The Hall effect was investigated by applying the four-probe method in constant electric and magnetic fields (*H* = 0.9 T) and current through a sample of 10 mA. The speed of sound was measured at *T* = 298 K using the ultrasonic flaw detector Olympus Panametrics Epoch 3.

### 4.4 Theoretical Calculations

Electronic structure calculations and bonding analyses were carried out for the ordered models without split positions with the compositions Na_2_Ga_6_Te_10_, Na_1.5_Ag_0.5_Ga_6_Te_10_, and Na_0.5_Ag_1.5_Ga_6_Te_10_ employing the TB-LMTO-ASA program package.^67,68^ The experimentally found symmetry and unit cell of the NaAgGa_6_Te_10_ sample and the atomic coordinates of the Na_2_Ga_6_Te_10_^32^ were used for the calculations. Sodium was placed at the 9*d* site (*xx*0) and silver was placed at the 3*b* site (001/2) for the model Na_1.5_Ag_0.5_Ga_6_Te_10_, and *vice versa* for the model Na_0.5_Ag_1.5_Ga_6_Te_10_. The Barth–Hedin exchange potential^69^ was employed for the LDA calculations. The radial scalar-relativistic Dirac equation was solved to obtain the partial waves. Despite the calculation within the atomic sphere approximation (ASA) including corrections for neglecting interstitial regions and partial waves of a higher order,^70^ an addition of empty spheres was necessary due to the loose character of the crystal structure with large voids within the Ga–Te network. The following radii of the atomic spheres were applied for the Na_1.5_Ag_0.5_Ga_6_Te_10_ model: *r*(Te1) = 1.617 Å, *r*(Te2) = 1.586 Å, *r*(Te3) = 1.608 Å, *r*(Te4) = 1.592 Å, *r*(Te5) = 1.669 Å, *r*(Ga1) = 1.399 Å, *r*(Ga2) = 1.418 Å, *r*(Ag) = 2.191 Å and *r*(Na) = 2.092 Å; the radii for the other two models are similar and can be obtained from the authors. A basis set containing Te(5s, 5p), Ga(4s, 4p), Ag(5s, 5p, 4d), and Na(3s) orbitals was employed for a self-consistent calculation with Te(5d,4f), Ga(4d), Ag(4f), and Na(3p, 3d) functions being downfolded. The analysis of the chemical bonding in position space^71,72^ was performed by means of the electron localizability approach. For this purpose, the electron localizability indicator (ELI) in its ELI-D representation^73,74^ and the electron density (ED) were calculated with a specialized module in the LMTO-ASA.^67,68^ The topologies of ELI-D and ED were evaluated by means of the program DGrid.^75^ The atomic charges from ED and bond populations for bonding basins from ELI-D were obtained by the integration of ED within the basins (space regions), bounded by zero-flux surfaces in the respective gradient field. This procedure follows the quantum theory of atoms in molecules (QTAIM).^76^

## Supporting Information Available

Thermal behavior; microstructure analysis; crystallographic information; conductivity activation energies; and details of the elastic and thermal transport properties calculations (PDF)
