# Supplementary material for: Lone-Pair-Like Interaction and Bonding Inhomogeneity Induce Ultralow Lattice Thermal Conductivity in Filled β-Manganese-Type Phases
Source: Chem Mater. 2022 May 27;34(14):6389–401. doi: 10.1021/acs.chemmater.2c00915 (PMC9344398; doi:10.1021/acs.chemmater.2c00915)
Supplement: Supplementary file 1 — cm2c00915_si_001.pdf [file cm2c00915_si_001.pdf]

Supporting Information

Lone-pair-like interaction and bonding inhomogeneity induce ultra-low lattice thermal conductivity in filled β–manganese-type phases

Oleksandr Cherniushok[1], Raul Cardoso-Gil[2], Taras Parashchuk[1], Rafal Knura[1,3], Yuri Grin[2]*, Krzysztof T. Wojciechowski[1]*

[1]Thermoelectric Research Laboratory, Department of Inorganic Chemistry, Faculty of Materials Science and Ceramics, AGH University of Science and Technology, Mickiewicza Ave. 30, 30-059 Krakow, Poland
[2]Max-Planck-Institut für Chemische Physik fester Stoffe, Nöthnitzer Str. 40, 01187 Dresden, Germany
[3]Department of Science, Graduate School of Science and Technology, Kumamoto University, 2 Chome-39-1 Kurokami, Chuo Ward, 860-8555 Kumamoto, Japan
*email: grin@cpfs.mpg.de; wojciech@agh.edu.pl

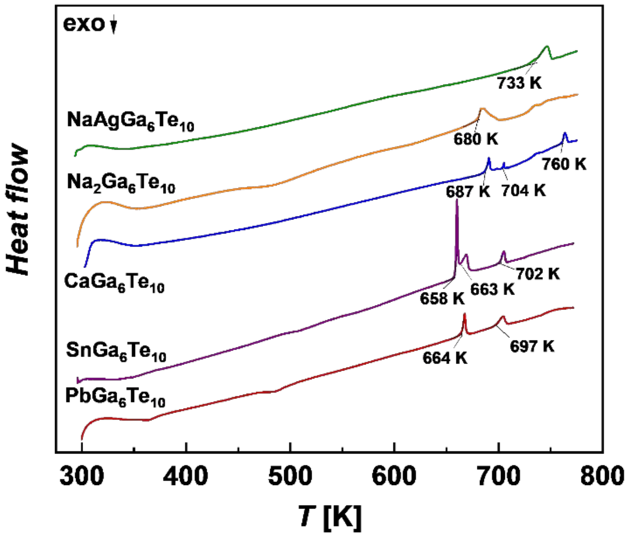

Figure S1. Thermal behavior of filled β-Mn-type phases.

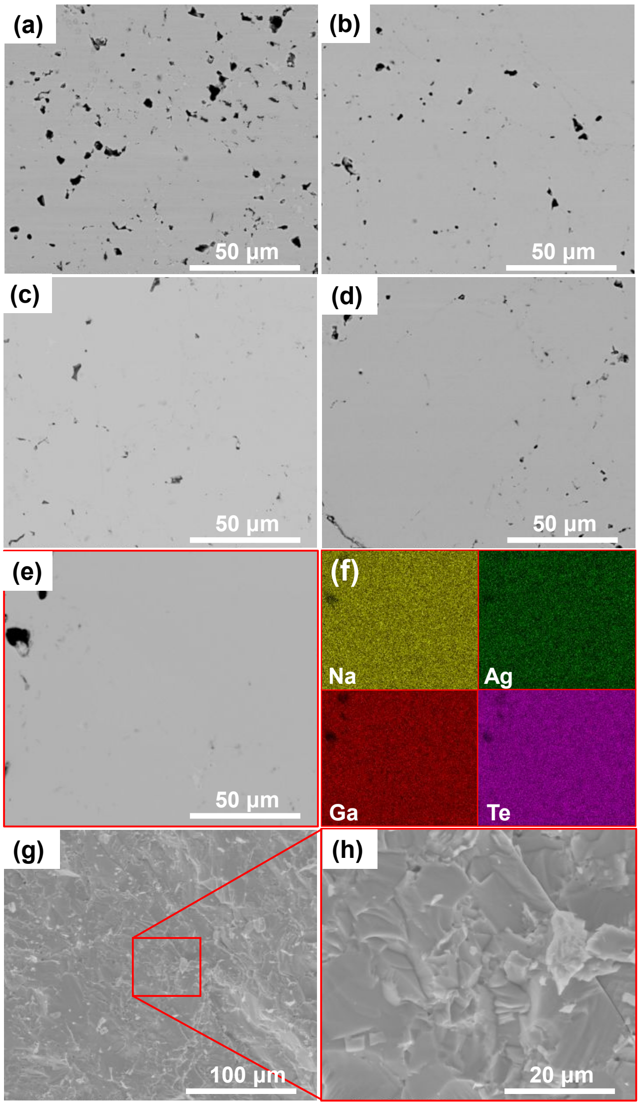

Figure S2. Microstructure (SEM images) for $CaGa_6Te_{10}$ (a), $SnGa_6Te_{10}$ (b), $PbGa_6Te_{10}$ (c), $Na_2Ga_6Te_{10}$ (d) and $NaAgGa_6Te_{10}$ (e) samples after SPS treatment; (f) EDXS element mapping

distribution for $NaAgGa_6Te_{10}$. Fractured surface of the $NaAgGa_6Te_{10}$ pellet after SPS treatment with lower (g) and higher (h) magnification.

Table S1. Atomic coordinates and isotropic displacement parameters for $CaGa_6Te_{10}$

Atom	Site	*x/a*	*y/b*	*z/c*	B_{eq} [Å^2]	Occupancy
Te1	18*f*	0.09975(9)	0.2990(1)	0.23029(8)	1.163(10)	1
Te2	18*f*	0.1863(1)	0.1283(2)	0.38008(8)	1.38(1)	1
Te3	9*e*	0.5824(2)	0	1/2	1.25(2)	1
Te4	9*d*	0.1702(2)	0	0	1.72(2)	1
Te5	6*c*	0	0	0.1708(2)	1.11(2)	1
Ga1	18*f*	0.0848(2)	0.3399(2)	0.0866(2)	1.41(5)	1
Ga2	18*f*	0.1883(2)	0.0773(2)	0.2387(2)	1.67(6)	1
Ca1	9*d*	0.7416(7)	0	0	1.17(6)	0.667

Table S2. Atomic coordinates and isotropic displacement parameters for $SnGa_6Te_{10}$

Atom	Site	*x/a*	*y/b*	*z/c*	B_{eq} [Å^2]	Occupancy
Te1	6*c*	0.1941(3)	0.5650(3)	0.0635(2)	1.15(8)	1
Te2	6*c*	0.9663(3)	1.1005(3)	-0.0615(2)	0.48(6)	1
Te3	6*c*	0.4775(3)	0.9439(3)	-0.2066(2)	0.88(6)	1
Te4	6*c*	0.6691(4)	1.0023(4)	-0.00102(9)	0.69(4)	1
Te5	6*c*	0.4645(3)	0.6963(3)	-0.0632(2)	0.84(6)	1
Te6	3*b*	0.8437(3)	0	1/6	1.17(10)	1
Te7	6*c*	0.7184(3)	0.8692(3)	-0.2121(2)	0.88(7)	1
Te8	6*c*	0.2118(3)	0.3875(3)	-0.1182(2)	1.35(7)	1
Te9	3*a*	0.7485(4)	0.7485(4)	0	1.16(11)	1
Te10	6*c*	0.3408(3)	0.9189(3)	0.0075(2)	1.23(5)	1
Te11	6*c*	0.1672(2)	0.6605(3)	-0.1699(2)	0.92(4)	1
Ga1	6*c*	0.2529(5)	0.5747(5)	-0.0816(4)	1.41(13)	1
Ga2	6*c*	0.4725(5)	0.8778(4)	-0.0653(3)	0.73(8)	1
Ga3	6*c*	0.4181(5)	0.6639(5)	0.0809(3)	0.99(12)	1
Ga4	6*c*	0.7814(5)	0.9226(5)	-0.0708(4)	0.76(14)	1
Ga5	6*c*	0.1871(5)	0.7476(5)	0.0759(3)	1.10(12)	1
Ga6	6*c*	0.9122(5)	0.9302(4)	-0.2523(3)	1.01(12)	1
Sn1	6*c*	0.9155(2)	1.2604(2)	0.1551(2)	1.50(4)	1

Table S3. Atomic coordinates and isotropic displacement parameters for $PbGa_6Te_{10}$

Atom	Site	*x/a*	*y/b*	*z/c*	B_{eq} [Å^2]	Occupancy
Te1	6*c*	0.1974(3)	0.5661(3)	0.0623(2)	0.85(3)	1
Te2	6*c*	0.9651(3)	1.1020(2)	-0.0618(2)	1.12(6)	1
Te3	6*c*	0.4826(3)	0.9450(3)	-0.2082(2)	1.25(6)	1
Te4	6*c*	0.6709(2)	1.0034(3)	-0.0019(1)	0.68(4)	1
Te5	6*c*	0.4665(3)	0.6982(3)	-0.0625(2)	1.09(6)	1
Te6	3*b*	0.8431(3)	0	1/6	1.08(9)	1
Te7	6*c*	0.7190(3)	0.8722(2)	-0.2112(2)	1.20(7)	1
Te8	6*c*	0.2119(2)	0.3901(3)	-0.1199(2)	1.25(7)	1
Te9	3*a*	0.7519(3)	0.7519(3)	0	1.03(10)	1
Te10	6*c*	0.3421(2)	0.9176(2)	0.0092(2)	1.21(4)	1
Te11	6*c*	0.1675(2)	0.6619(2)	-0.1708(3)	1.46(6)	1
Ga1	6*c*	0.2487(4)	0.5743(5)	-0.0843(4)	1.43(11)	1
Ga2	6*c*	0.4788(4)	0.8841(4)	-0.0629(4)	0.97(11)	1
Ga3	6*c*	0.4114(4)	0.6596(4)	0.0779(4)	1.08(9)	1
Ga4	6*c*	0.7820(4)	0.9243(4)	-0.0707(4)	0.54(12)	1
Ga5	6*c*	0.1865(4)	0.7446(4)	0.0756(3)	0.32(10)	1
Ga6	6*c*	0.9156(4)	0.9274(4)	-0.2523(3)	1.46(8)	1
Pb1	6*c*	0.9201(2)	1.2617(2)	0.1587(2)	1.98(2)	1

Table S4. Atomic coordinates and isotropic displacement parameters for $Na_2Ga_6Te_{10}$ (ordered model)

Atom	Site	*x/a*	*y/b*	*z/c*	B_{eq} [Å^2]	Occupancy
Te1	6*c*	0	0	0.1720(2)	1.40(7)	1
Te2	9*d*	0.1726(2)	0.1726(2)	0	1.34(5)	1
Te3	18*f*	0.5674(1)	0.0345(1)	0.1036(2)	1.02(3)	1
Te4	9*e*	1/3	0.0893(2)	1/6	1.33(5)	1
Te5	18*f*	0.1903(2)	0.0609(2)	0.3775(1)	1.22(3)	1
Ga1	18*f*	0.5840(2)	-0.0061(3)	0.2485(3)	1.08(6)	1
Ga2	18*f*	0.1880(3)	0.1143(2)	0.2347(3)	1.12(7)	1
Na1	3*b*	0	0	1/2	1.3(5)	1
Na2	9*d*	0.2600(10)	0	0	2.4(3)	1

Table S5. Atomic coordinates and isotropic displacement parameters for $Na_2Ga_6Te_{10}$ (disordered model)

Atom	Site	*x/a*	*y/b*	*z/c*	B_{eq} [Å^2]	Occupancy
Te1	6*c*	0	0	0.1721(2)	1.50(3)	1
Te2	9*d*	0.1732(2)	0.1732(2)	0	1.44(5)	1
Te3	18*f*	0.5675(1)	0.0343(1)	0.1036(2)	0.99(3)	1
Te4	9*e*	1/3	0.0892(2)	1/6	1.32(5)	1
Te5	18*f*	0.1902(2)	0.0609(2)	0.3775(1)	1.20(3)	1
Ga1	18*f*	0.5846(2)	-0.0058(3)	0.2484(2)	1.15(2)	1
Ga2	18*f*	0.1877(2)	0.1141(2)	0.2347(3)	1.15(6)	1
Na1	3*b*	0	0	1/2	0.7(5)	1
Na2	9*d*	0.260(2)	0	0	1.0(5)	0.48(2)
Na3	18*f*	0.331(2)	-0.028(3)	0.029(2)	1.000	0.26(1)
Na4	9*d*	0.256(8)	0	0	1.0(23)	0.12(2)
Na5	18*f*	0.417(4)	0.059(4)	0.311(4)	1.000	0.147(9)

Table S6. Atomic coordinates and isotropic displacement parameters for $NaAgGa_6Te_{10}$ (PXRD)

Atom	Site	*x/a*	*y/b*	*z/c*	B_{eq} [Å^2]	Occupancy
Te1	6*c*	0	0	0.1707(2)	1.12(6)	1
Te2	9*d*	0.1673(2)	0.1673(2)	0	1.75(5)	1
Te3	18*f*	0.5673(1)	0.0338(2)	0.1028(1)	1.16(2)	1
Te4	9*e*	1/3	0.0847(3)	1/6	1.53(5)	1
Te5	18*f*	0.1913(2)	0.0579(2)	0.3766(1)	1.28(2)	1
Ga1	18*f*	0.5849(3)	0.0003(3)	0.2483(2)	1.45(5)	1
Ga2	18*f*	0.1901(2)	0.1129(2)	0.2359(2)	0.40(5)	1
Na1	9*d*	0.2777(7)	0	0	1.4(2)	0.304 Na + 0.161 Ag
Na2	18*f*	0.3222(14)	0.018(2)	0.010(2)	1.4(4)	0.116 Na + 0.061 Ag
Na3	3*b*	0	0	1/2	2.0(7)	0.100 Na + 0.065 Ag
Na4	9*d*	0.231(2)	0	0	0.9(5)	0.130 Na + 0.068 Ag
Na5	18*f*	0.416(3)	0.082(3)	0.311(2)	1.2(6)	0.086 Na + 0.045 Ag

Table S7. Atomic coordinates and isotropic displacement parameters for $NaAgGa_6Te_{10}$

(Single crystal XRD)

Atom	Site	*x/a*	*y/b*	*z/c*	B_{eq} [Å^2]	Occupancy
Te1	6*c*	0	0	0.1700(2)	3.02(5)*	1
Te2	9*d*	0.1687(2)	0.1687(2)	0	3.67(6)*	1
Te3	18*f*	0.5670(1)	0.0327(1)	0.10313(8)	3.07(4)*	1
Te4	9*e*	1/3	0.0854(2)	1/6	3.75(6)*	1
Te5	18*f*	0.1919(2)	0.0580(1)	0.37690(9)	3.45(5)*	1
Ga1	18*f*	0.5839(2)	-0.0030(2)	0.2484(2)	3.28(8)*	1
Ga2	18*f*	0.1896(2)	0.1133(2)	0.2364(2)	3.28(8)*	1
Na1	9*d*	0.2771(11)	0	0	2.000	0.304 Na + 0.161 Ag
Na2	18*f*	0.316(2)	0.002(2)	0.0156(9)	2.000	0.116 Na + 0.061 Ag
Na3	3*b*	0	0	1/2	2.000	0.100 Na + 0.065 Ag
Na4	9*d*	0.236(2)	0	0	2.000	0.130 Na + 0.068 Ag
Na5	18*f*	0.408(2)	0.082(2)	0.3077(14)	2.000	0.086 Na + 0.045 Ag

*Anisotropic displacement parameters for $NaAgGa_6Te_{10}$

Atom	B_{11}	B_{22}	B_{33}	B_{12}	B_{13}	B_{23}
Te1	2.95(6)	B_{11}	3.15(8)	$1/2B_{11}$	0	0
Te2	3.40(6)	B_{11}	3.61(8)	1.24(7)	0.20(4)	- B_{13}
Te3	3.06(6)	3.07(6)	3.09(5)	1.54(5)	-0.03(4)	0.02(4)
Te4	3.77(10)	3.25(6)	4.40(9)	$1/2B_{11}$	0.94(8)	$1/2B_{13}$
Te5	3.64(7)	3.10(6)	3.31(5)	1.46(5)	-0.05(5)	0.04(5)
Ga1	3.37(10)	3.47(10)	3.05(8)	1.76(9)	0.06(7)	-0.07(7)
Ga2	3.19(10)	3.18(9)	3.38(9)	1.53(8)	-0.06(8)	-0.05(7)

Table S8. Comparison between the estimated activation energies E_a in the given temperature range with the bandgap values E_g for studied filled β-manganese-type phases

Compound	E_a [eV] / temp. range [K]	Experimental E_g, [eV]
**$CaGa_6Te_{10}$**	0.59(4) / 298-495,<br>0.83(1) / 495-646<br>1.08(3) / 671-747	-
**$SnGa_6Te_{10}$**	0.77(2) / 298-495,<br>0.82(5) / 495-651,<br>1.01(1) / 696-773	1.25(1) [1]<br>(E_a = 0.90 [1])
**$PbGa_6Te_{10}$**	0.56(3) / 298-495,<br>1.43(3) / 495-620<br>1.35(3) / 648-773	1.35(1) [1]<br>(E_a = 0.78 [1])
**$Na_2Ga_6Te_{10}$**	0.52(3) / 298-495<br>1.53(11) / 495-620<br>1.65(1) / 645-773	-
**$NaAgGa_6Te_{10}$**	0.41(2) / 298-495<br>0.95(3) / 495-671<br>0.78(7) / 696-773	0.9 [this work, DFT]

Elastic properties

The bulk modulus was calculated using the following equation [2]:

$$B = \rho\left(\upsilon_L^2 - \frac{4}{3}\upsilon_T^2\right), \tag{S1}$$

where ρ is the material density.

The shear modulus was calculated as:

$$G = \upsilon_T^2 \rho. \tag{S2}$$

The Young's modulus is calculated as:

$$E = \frac{9BG}{3B+G}. \tag{S3}$$

The Poisson's ratio is calculated as:

$$\nu = \frac{E-2G}{2G}. \tag{S4}$$

The Debye temperatures were calculated using the following expression [3]:

$$\Theta_D = \frac{h}{k_B}\left[\frac{3n}{4\pi}\left(\frac{N_A\rho}{M}\right)\right]^{1/3} v_m , \tag{S5}$$

where h is Planck's constant, k_B is Boltzmann's constant, N_A is Avogadro's number, M is the molecular weight, n is the number of atoms in the molecule, and v_m is the averaged wave velocity integrated over several crystal directions [3]:

$$v_m = \left[\frac{1}{3}\left(\frac{2}{v_t^3} + \frac{1}{v_l^3}\right)\right]^{-1/3} , \tag{S6}$$

where v_l and v_t are the longitudinal and transverse sound velocities, respectively. Grüneisen parameters γ were calculated using the following formula [4]:

$$\gamma = \frac{3}{2}\left(\frac{1+\nu}{2-3\nu}\right). \tag{S7}$$

Thermal transport properties

The phonon mean free paths were calculated by [5]:

$$l_{ph} = \frac{3\kappa_{lat}}{C_V v_m}. \tag{S8}$$

Considering the ultrasonic data, the lattice thermal conductivity was also calculated as follows [6]:

$$\kappa_L = K\frac{\bar{M}\Theta_D^3\delta}{\gamma^2 n^{\frac{2}{3}} T}. \tag{S9}$$

Here n is the number of atoms in the primitive unit cell, δ^3 is the volume per atom, Θ_D is the Debye temperature, $\bar{M}$ is the average mass of the atoms in the crystal, and C is a collection of physical constants ($K \approx 3.1\times10^{-6}$ if κ_L is in $Wm^{-1}K^{-1}$, $\bar{M}$ in amu, and δ in Angstroms).

According to Cahill's formulation based on the maximum phonon scattering approach, the glassy limit for the thermal conductivity κ_{glass} was estimated by [7]:

$$\kappa_{glass} = \frac{1}{2}\left(\frac{\pi}{6}\right)^{1/3} k_B V^{-2/3}\left(2v_t + v_l\right), \tag{S10}$$

where V is the average volume per atom calculated from the refined lattice parameters. The diffusion-based minimum of the thermal conductivity κ_{diff} was calculated using the Cahill plot, as proposed by Agne et al. [8] expression:

$$\kappa_{diff} \approx 0.76 n^{2/3} k_B \frac{1}{3}\left(2v_t + v_l\right) \approx 0.63\kappa_{glass}. \tag{S11}$$

The phonon relaxation time (τ_c) is calculated using contributions related to point defects scattering (τ_p) [9], phonon-phonon Umklapp scattering (τ_U) [10,11], grain boundary scattering (τ_B), and phonon resonance scattering (τ_R) [12]:

$$\tau_P^{-1} = A\left(\frac{k_B T}{\hbar}\right)^4 t^4, \tag{S12}$$

$$\tau_U^{-1} = B\left(\frac{k_B T}{\hbar}\right)^2 e^{\left(\frac{-\Theta_D}{3T}\right)} T t^2, \tag{S13}$$

$$\tau_B^{-1} = \frac{v_m}{d}, \tag{S14}$$

$$\tau_R^{-1} = \frac{CH\omega^2}{(\omega_0^2 - \omega^2)^2 + (\Lambda/\pi)^2 \omega_0^2 \omega^2}, \tag{S15}$$

where, $\hbar = h/(2\pi)$, $t = \hbar\omega/(k_B T)$, *A, B, and C* are adjustable fitting parameters related to point defect scattering, phonon-phonon Umklapp scattering processes, and resonance phonon scattering, respectively. d is the grain size, H is the half-width of the energy resonance curve ($H = \omega_0 \Lambda / 2\pi^2$), C is a proportionality factor containing the concentration of oscillators, ω_0 is the resonance circular frequency, and Λ is the logarithmic decrement [$\Lambda = (2Q)^{-1}$].

(1) Duguzhev, S. M.; Makhin, A. V.; Moshnikov, V. A.; Shelykh, A. I. Doping of PbTe and Pb1−xSnxTe with Gallium and Indium. *Cryst. Res. Technol.* **1990,** *25* (2), 145–149. https://doi.org/10.1002/crat.2170250208.

(2) Papadakis, E. P.; Papdakis, E. P.; Stickels, C. A.; Innes, R. C. An Ultrasonic Technique for Measuring the Elastic Constants of Small Samples. *SAE Trans.* **1995**, *104*, 830–837.

(3) Anderson, O. L. A Simplified Method for Calculating the Debye Temperature from Elastic Constants. *J. Phys. Chem. Solids* **1963**, *24* (7), 909–917.

https://doi.org/10.1016/0022-3697(63)90067-2.
(4) Sanditov, D. S.; Belomestnykh, V. N. Relation between the Parameters of the Elasticity Theory and Averaged Bulk Modulus of Solids. *Tech. Phys.* **2011**, *56* (11), 1619–1623. https://doi.org/10.1134/S106378421111020X.
(5) Xie, H.; Hao, S.; Cai, S.; Bailey, T. P.; Uher, C.; Wolverton, C.; Dravid, V. P.; Kanatzidis, M. G. Ultralow Thermal Conductivity in Diamondoid Lattices: High Thermoelectric Performance in Chalcopyrite Cu0.8+yAg0.2In1−yTe2. *Energy Environ. Sci.* **2020**, *13* (10), 3693–3705. https://doi.org/10.1039/D0EE02323J.
(6) Morelli, D. T.; Jovovic, V.; Heremans, J. P. Intrinsically Minimal Thermal Conductivity in Cubic I-V-VI2 Semiconductors. *Phys. Rev. Lett.* **2008**, *101* (3), 035901. https://doi.org/10.1103/PhysRevLett.101.035901.
(7) Cahill, D. G.; Pohl, R. O. Lattice Vibrations and Heat Transport in Crystals and Glasses. *Annu. Rev. Phys. Chem.* **1988**, *39* (1), 93–121. https://doi.org/10.1146/annurev.pc.39.100188.000521.
(8) Agne, M. T.; Hanus, R.; Snyder, G. J. Minimum Thermal Conductivity in the Context of: Diffuson -Mediated Thermal Transport. *Energy Environ. Sci.* **2018**, *11* (3), 609–616. https://doi.org/10.1039/c7ee03256k.
(9) Callaway, J. Model for Lattice Thermal Conductivity at Low Temperatures. *Phys. Rev.* **1959**, *113* (4), 1046–1051. https://doi.org/10.1103/PhysRev.113.1046.
(10) Glassbrenner, C. J.; Slack, G. A. Thermal Conductivity of Silicon and Germanium from 3°K to the Melting Point. *Phys. Rev.* **1964**, *134* (4A), A1058. https://doi.org/10.1103/PhysRev.134.A1058.
(11) Slack, G. A.; Galginaitis, S. Thermal Conductivity and Phonon Scattering by Magnetic Impurities in CdTe. *Phys. Rev.* **1964**, *133* (1A), A253. https://doi.org/10.1103/PhysRev.133.A253.
(12) Pohl, R. O. Thermal Conductivity and Phonon Resonance Scattering. *Phys. Rev. Lett.* **1962**, *8* (12), 481. https://doi.org/10.1103/PhysRevLett.8.481.
